# Supplementary material for: Dental Caries and the Erosive Tooth Wear Status of 12-Year-Old Children in Jakarta, Indonesia
Source: Int J Environ Res Public Health. 2019 Aug 20;16(16):2994. doi: 10.3390/ijerph16162994 (PMC6720660; doi:10.3390/ijerph16162994)
Supplement: Supplementary file 1 [file ijerph-16-02994-s001.pdf]

Oral Health Questionnaire

Child name :

Gender : □Male □Female

Place and date of birth:

Class and School:

Caregiver :

Contact number (for further follow-up):

The educational level of the parents who live with the child:

	Father	Mother
Primary or lower	□	□
Secondary	□	□
College or higher	□	□

How often does your child brush his/her teeth?

□ Never / irregularly

□ Once a day

□ Twice a day

□ Three times a day or more

Part A: Dietary habit of your child

1) How often does your child have soft drinks?

□ 6, 7 times a week or more (almost every day or more)

□ 3 times a week (alternate days)

□ Once a week

□ Less than once a week / never

2) How often does your child have citric tea / drinks containing lemon?

□ 6, 7 times a week or more (almost every day or more)

□ 3 times a week (alternate days)

□ Once a week

□ Less than once a week / never

3) How often does your child drink fruit juice?

□ 6, 7 times a week or more (almost every day or more)

□ 3 times a week (alternate days)

□ Once a week

□ Less than once a week / never

4) How often does your child have vitamin C supplement drinks?

□ 6, 7 times a week or more (almost every day or more)

□ 3 times a week (alternate days)

□ Once a week

□ Less than once a week / never

5) How often does your child have chewing gum?

- □ 6, 7 times a week or more (almost every day or more)
- □ 3 times a week (alternate days)
- □ Once a week
- □ Less than once a week / never

Pert B: Parent's oral health knowledge

	Yes	No	Don't know
1) The causes of dental decay include:			
a) Too much consumption of candies	□	□	□
b) Unclean teeth	□	□	□
c) Tooth worms attack	□	□	□
d) "Hot air"	□	□	□
2) Preventions of tooth decay include:			
a) Using miswak	□	□	□
b) Gargle with salted water	□	□	□
c) Use of fluoridated toothpaste	□	□	□
d) Decrease frequency of sugar consumption	□	□	□
3) Effects of fluoride to teeth include:			
a) No effect	□	□	□
b) Prevent tooth decay	□	□	□
c) Tooth whitening	□	□	□
d) Prevent periodontal disease	□	□	□
4) Which of the following food can cause tooth decay?			
a) Soft drinks	□	□	□
b) Ice-cream	□	□	□
c) Cheese	□	□	□
d) Peanuts	□	□	□
5) The causes of gum bleeding include:			
a) Unclean teeth	□	□	□
b) It Is a normal phenomenon	□	□	□
6) Methods to prevent periodontal disease include:			
a) Tooth brushing	□	□	□
b) Saline mouth-rinsing	□	□	□
c) Regular scaling (professional tooth cleaning)	□	□	□
